# Supplementary material for: Lipid nanodisc scaffold and size alter the structure of a pentameric ligand-gated ion channel
Source: Nat Commun. 2024 Jan 2;15:25. doi: 10.1038/s41467-023-44366-w (PMC10762164; doi:10.1038/s41467-023-44366-w)
Supplement: Supplementary file 1 — Supplementary Information [file 41467_2023_44366_MOESM1_ESM.pdf]

Lipid nanodisc scaffold and size alter the structure of a pentameric ligand-gated ion channel

Vikram Dalal[1,†], Mark J. Arcario[1,†], John T. Petroff II[1], Brandon K. Tan[1], Noah M. Dietzen[1], Michael J. Rau[2], James A. J. Fitzpatrick[2], Grace Brannigan[3,4], Wayland W. L. Cheng[1*]

[1]Department of Anesthesiology, Washington University School of Medicine, Saint Louis, MO, USA

[2]Center for Cellular Imaging, Washington University School of Medicine, Saint Louis, MO, USA

[3]Center for Computational and Integrative Biology, Rutgers University, Camden, NJ, USA

[4]Department of Physics, Rutgers University, Camden, NJ, USA

† authors contributed equally to this manuscript

*To whom correspondence should be addressed: Professor Wayland W. L. Cheng, Department of Anesthesiology, Washington University School of Medicine, MSC 8054-0043-12, Saint Louis, MO 63110. Telephone: (314)273-7958; E-mail: wayland.cheng@wustl.edu

Supplementary Table 1: Cryo-EM data collection and refinement statistics

	SMA_{ELIC} EMD-28829 PDB 8F32	$saposin_{ELIC}$ EMD-28830 PDB 8F33	$spMSP1D1_{ELIC}$ EMD-28831 PDB 8F34	apo-$spMSP1D1_{ELIC}$ EMD-28832 PDB 8F35	$spNW15_{ELIC}$ EMD-41673 PDB 8TWZ	$spNW15_{ELIC5}$ EMD-41672 PDB 8TWV
**Data collection and processing**						
Magnification	75000	96000	96000	96000	59000	120000
Voltage (kV)	300	300	300	300	300	200
Electron exposure (e-/ Å^2)	49.45	47.55	46.93	51.54	54.6	50.09
Defocus range (μm)	-0.8 - 2.4	-0.8 - 2.4	-0.8 - 2.4	-0.8 - 2.4	-0.8 – 2.4	-1 -2.4
Pixel size (Å)	0.842	0.657	0.657	0.657	1.081	1.184
Symmetry imposed	C5	C5	C5	C5	C5	C5
Initial particle images (no)	1391288	1026975	1168080	837164	1560778	1719651
Final particle images (no)	128454	84696	65209	36562	58178	27201
Map resolution (Å)	3.71	3.28	3.12	3.17	3.17	3.40
FSC threshold	0.143	0.143	0.143	0.143	0.143	0.143
**Refinement**						
Initial model used	PDB 8F34	PDB 8F34	PDB 8D65	PDB 8F34	PDB 8F34	PDB 8D68
Model resolution (Å)	3.7	3.3	3.2	3.2	3.2	3.4
FSC threshold	0.5	0.5	0.5	0.5	0.5	0.5
Map sharpening B factor (Å^2)	-191.82	-144.03	-135.46	-144.96	-154.89	-178.5
**Model composition**						
Non-hydrogen atoms	12545	12545	12545	12525	11250	12720
Protein Residues	1535	1535	1535	1535	1370	1550
Ligands	5	5	5	0	5	5
**B factors (Å^2) (0.5)**						
Protein	61.31	47.17	31.14	21.72	41.36	81.24
Ligand	20.00	20.00	20.00	---------	19.74	20.00
**R.m.s. deviations**						
Bond lengths (Å)	0.005	0.006	0.005	0.005	0.005	0.005

Bond angles (°)	1.155	1.144	1.100	1.064	1.063	1.076
**Validation**						
MolProbity Score	1.89	1.61	1.80	2.01	1.80	1.58
Clashscore	12.13	6.249	6. 49	9.43	5.99	11.00
Poor rotamers (%)	0.00	0.00	0.00	0.00	0.00	0.00
**Ramachandran plot**						
Favored (%)	95.74	96.07	93.44	91.15	92.43	95.13
Allowed (%)	4.26	3.93	6.56	8.85	7.57	4.87
Disallowed (%)	0.00	0.00	0.00	0.00	0.00	0.00

Supplementary Table 2. Simulation system details for each independent run

Simulation Condition	Run	Total No. POPC	No. Upper Leaflet POPC	No. Lower Leaflet POPC	Total No. Atoms	Simulation Time (μs)
9 nm Nanodisc (MSP1D1-33)	1	53	23	30	363,764	0.5
	2	53	23	30	362,874	0.5
	3	53	23	30	363,762	0.5
11 nm Nanodisc (MSP1E2D1)	1	139	66	73	457,187	0.5
	2	139	66	73	461,603	0.5
	3	139	66	73	462,158	0.5
Planar Bilayer	1	265	129	136	158,847	0.5
	2	265	129	136	159,396	0.5
	3	265	129	136	159,387	0.5

Supplementary Figure 1

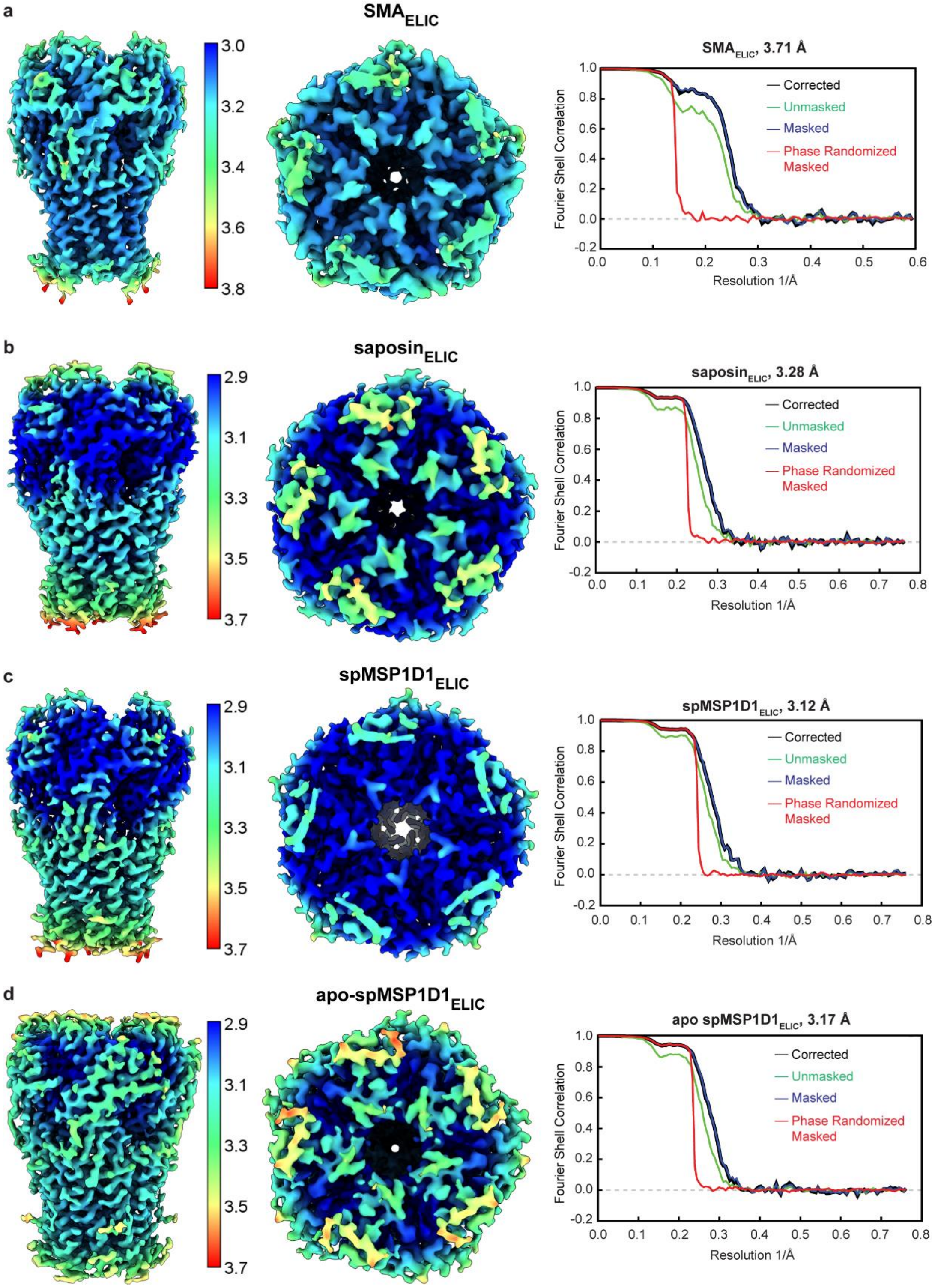

Supplementary Figure 1: Local resolution maps and fourier shell correlation (FSC) curves. Post-processed maps colored by local resolution and FSC curves generated by Relion for **(a)** SMA_{ELIC}, **(b)** $saposin_{ELIC}$, **(c)** $spMSP1D1_{ELIC}$, and **(d)** apo-$spMSP1D1_{ELIC}$. For each reconstruction, the local resolution was estimated by PHENIX using the sum of experimental half maps.

Supplementary Figure 2

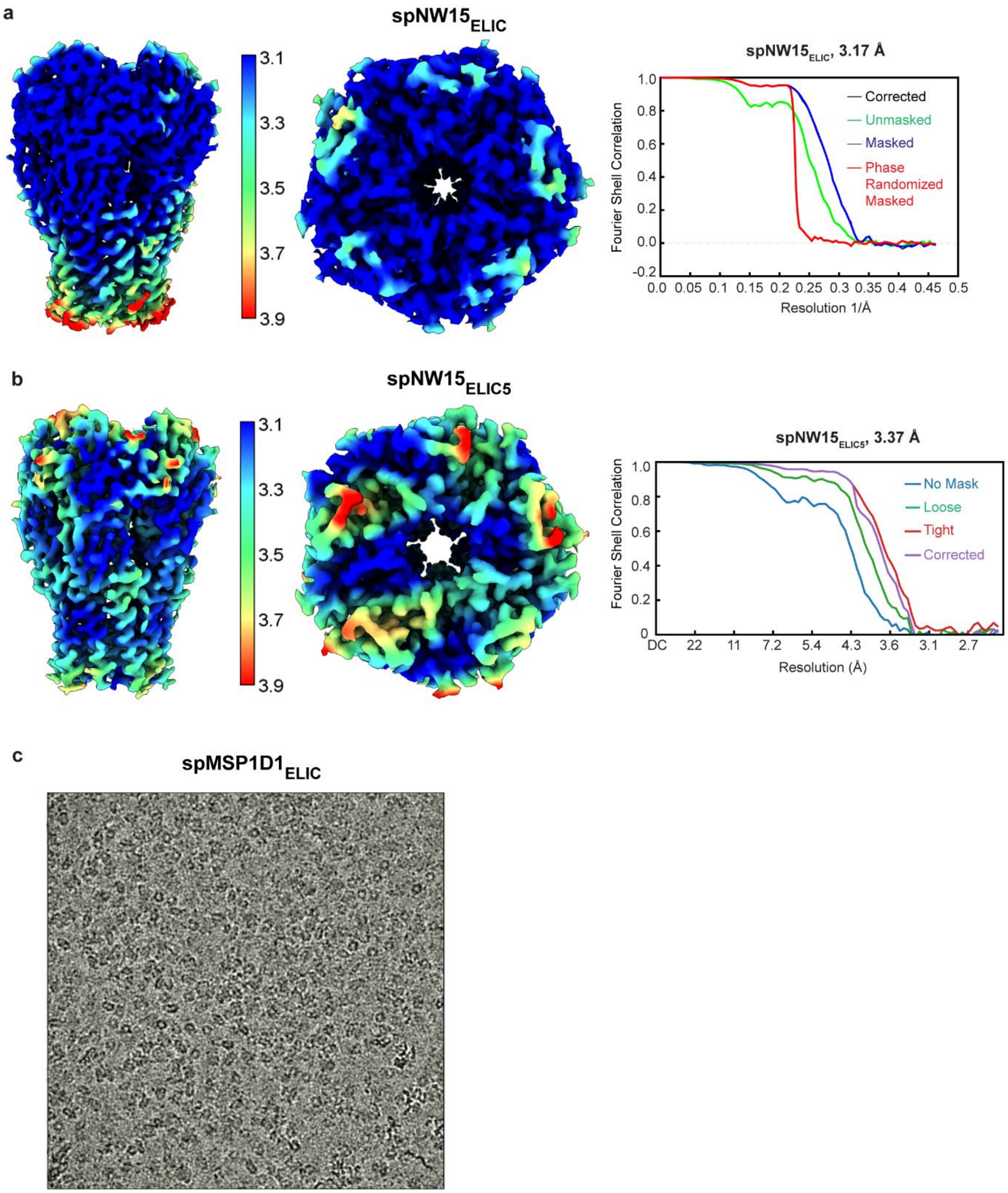

Supplementary Figure 2: Local resolution maps and FSC curves for spNW15$_{ELIC}$ and spNW15$_{ELIC5}$. Post-processed maps colored by local resolution for **(a)** spNW15$_{ELIC}$ **(b)** spNW15$_{ELIC5}$, generated by PHENIX using the sum of experimental half maps. FSC curves for spNW15$_{ELIC}$ (a) and spNW15$_{ELIC5}$ (b) were generated in Relion and CryoSPARC, respectively. **(c)** Representative micrograph from the spMSP1D1$_{ELIC}$ dataset.

Supplementary Figure 3

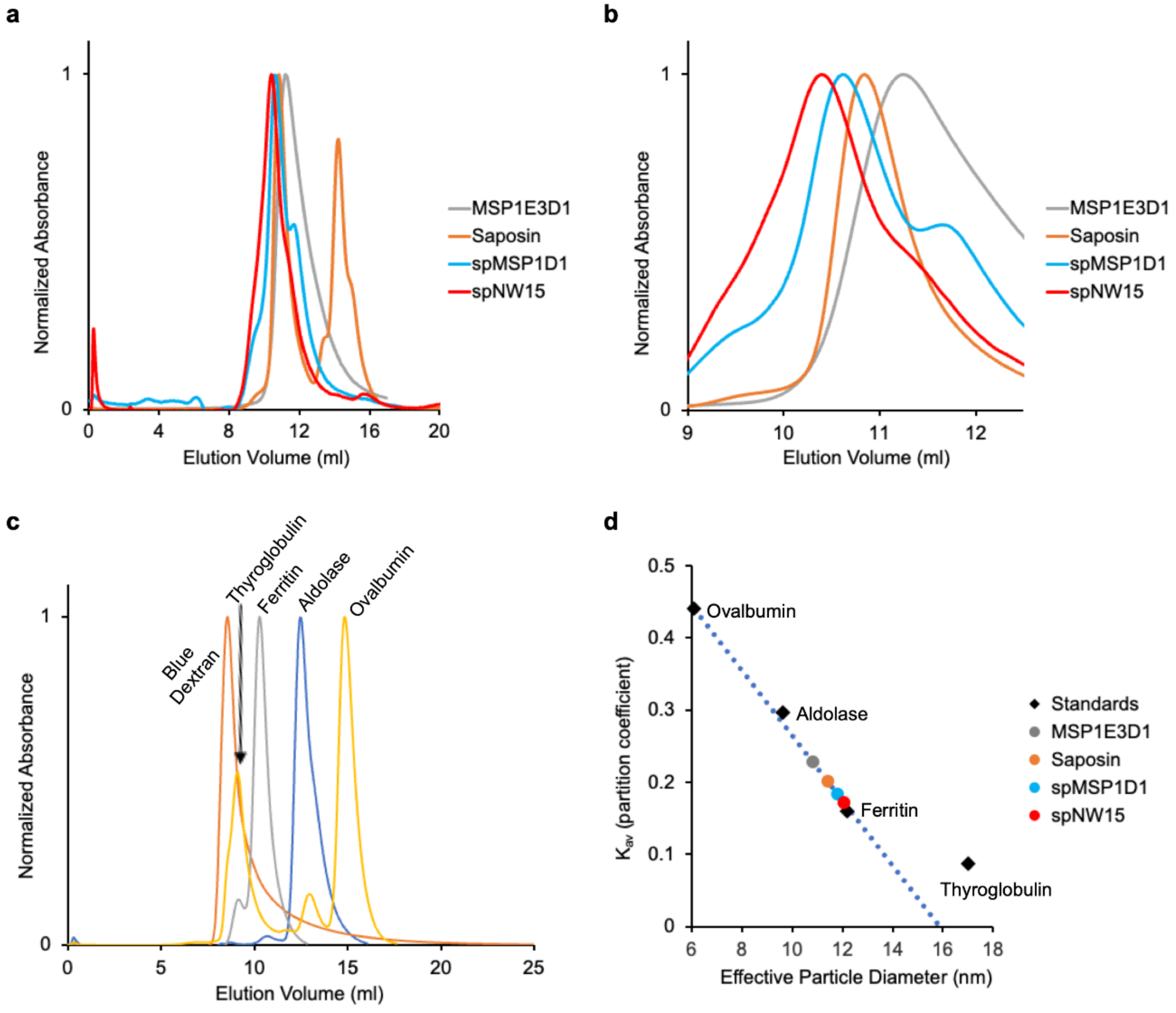

e

	MSP1E3D1	Saposin	spMSP1D1	spNW15
**Kav**	0.247	0.201	0.184	0.172
**Eff Diameter (nm)**	10.8	11.4	11.8	12.1

Supplementary Fig. 3: Size exclusion (SEC) analysis of ELIC in different nanodiscs. (a) and **(b)** SEC profiles of ELIC reconstituted in MSP1E3D1, saposin, spMSP1D1, and spNW15 on Superdex 200 Increase 10/300 GL. **(c)** SEC profile for standards: blue dextran, thyroglobulin, ferritin, aldolase, and ovalbumin. **(d)** Plot of partition coefficient (Kav) versus effective particle diameter (nm) for standards and ELIC in MSP1E3D1, saposin, spMSP1D1, and spNW15. **(e)** Estimated effective particle diameter (nm) for ELIC in the indicated nanodiscs.

Supplementary Figure 4

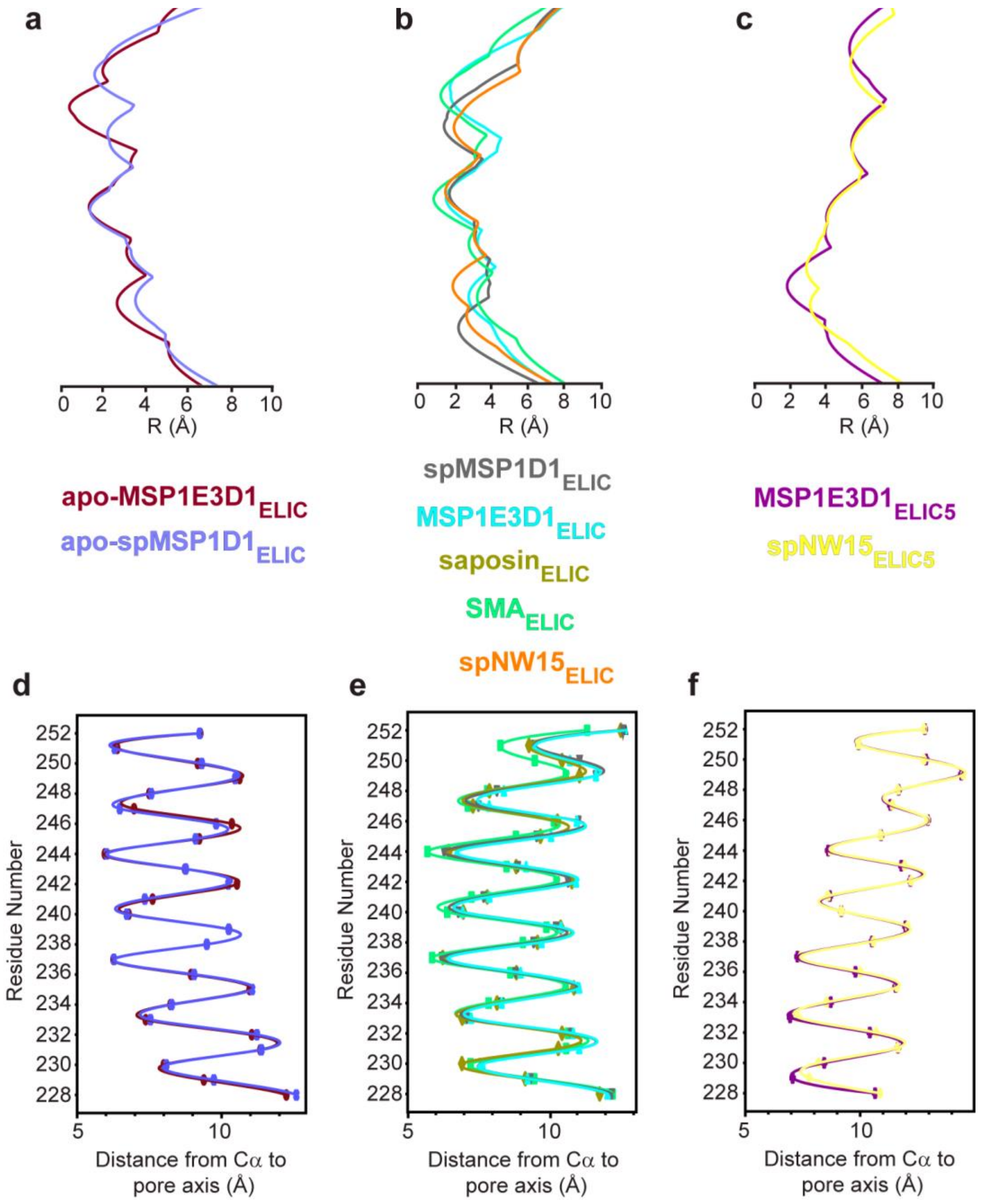

Supplementary Fig. 4: Pore profiles for ELIC structures. Comparison of the pore radius profile of **(a)** apo-MSP1E3D1$_{ELIC}$ and apo-spMSP1D1$_{ELIC}$, **(b)** spMSP1D1$_{ELIC}$, MSP1E3D1$_{ELIC}$, SMA$_{ELIC}$, and spNW15$_{ELIC}$ and **(c)** MSP1E3D1$_{ELIC5}$ and spNW15$_{ELIC5}$**.** Overlapping profiles are derived from a global superposition of all structures, and determined using HOLE. Shown below are plots of the M2 Cα-atom distance to the pore axis for **(d)** apo-MSP1E3D1$_{ELIC}$ and apo-spMSP1D1$_{ELIC}$, **(e)** spMSP1D1$_{ELIC}$, MSP1E3D1$_{ELIC}$, SMA$_{ELIC}$, and saposin$_{ELIC}$ and **(f)** MSP1E3D1$_{ELIC5}$ and spNW15$_{ELIC5}$**.** Solid lines are cubic-spline interpolations.

Supplementary Figure 5

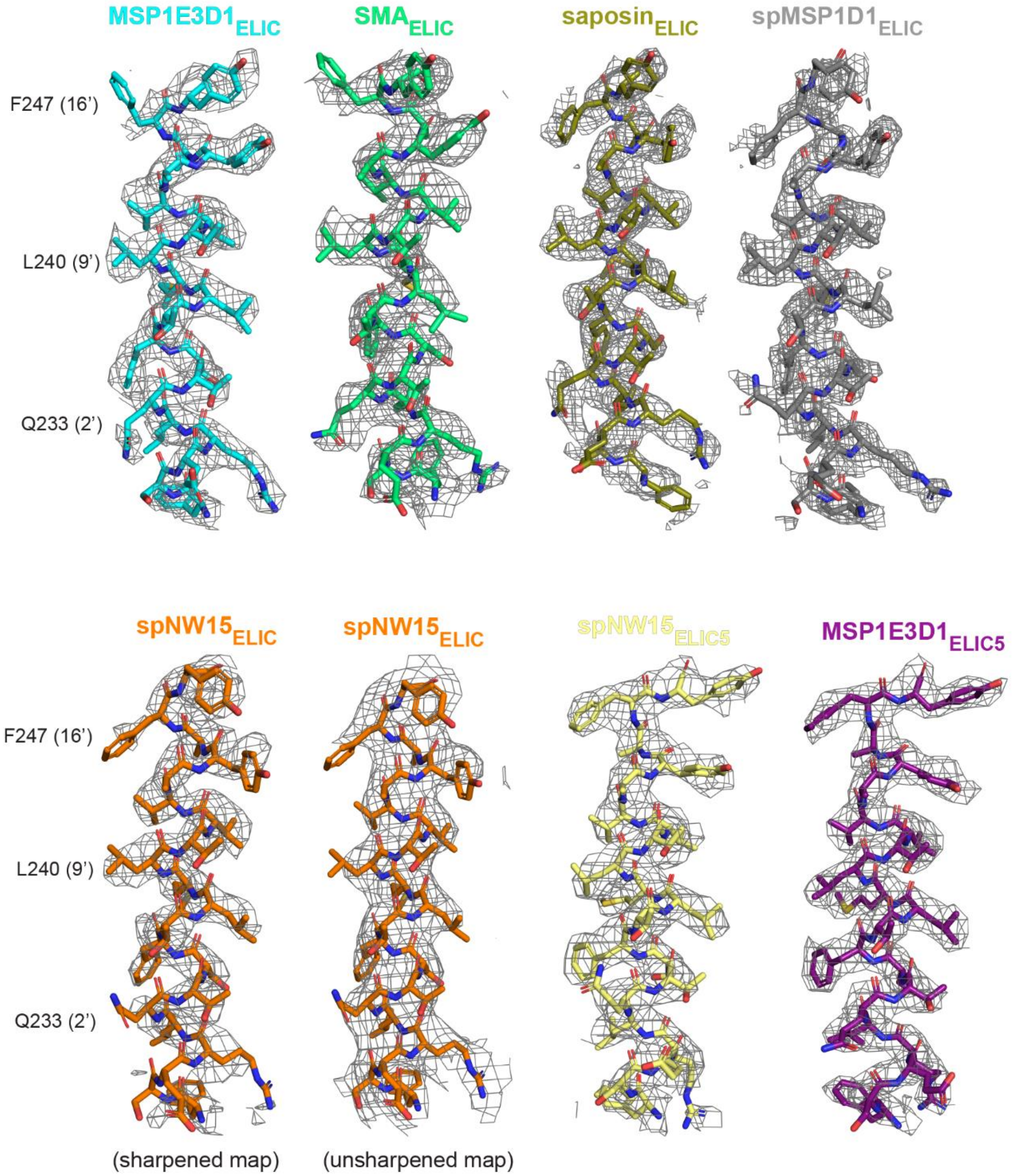

Supplementary Fig. 5: **Cryo-EM densities of M2.** Cryo-EM densities from sharpened maps with a contour level of 3.0 σ for MSP1E3D1$_{ELIC}$ and 3.5 σ for all other structures. Images were generated in PyMOL. To show optimal fitting of the bottom of M2 of spNW15$_{ELIC}$, both sharpened and unsharpened maps are shown. The weaker density in the sharpened map at the bottom of M2 in spNW15$_{ELIC}$ suggests some structural heterogeneity in this region. Side chains for F247 (16'), L240 (9'), and Q233 (2') are labeled.

Supplementary Figure 6

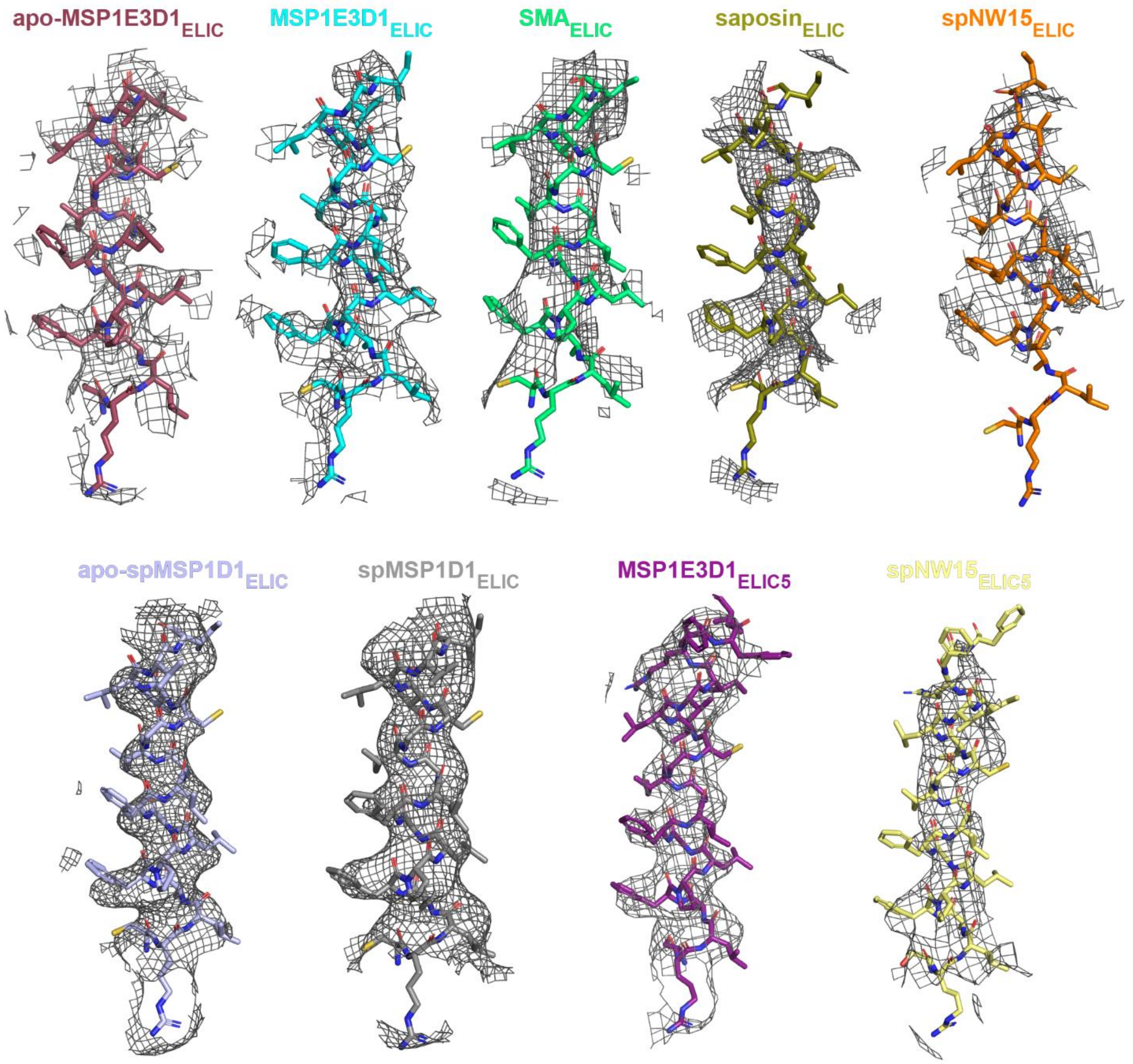

Supplementary Fig. 6: Cryo-EM densities of M4. Cryo-EM densities from unsharpened maps generated in PyMOL. The contour levels for each structure are 2.5 σ for apo-MSP1E3D1$_{ELIC}$, 2.5 σ for MSP1E3D1$_{ELIC}$, 2.8 σ for SMA$_{ELIC}$, 2.8 σ for saposin$_{ELIC}$, 2.0 σ for spNW15$_{ELIC}$, 3.0 σ for apo-spMSP1D1$_{ELIC}$, 3.0 σ for spMSP1D1$_{ELIC}$, 3.2 σ for MSP1E3D1$_{ELIC5}$, and 3.2 σ for spNW15$_{ELIC5}$. There is an absence of cryo-EM density for M4 in spNW15$_{ELIC}$ evidenced by the noisy signal at 2.0 σ; therefore, M4 was not included in the final model of spNW15$_{ELIC}$.

Supplementary Figure 7

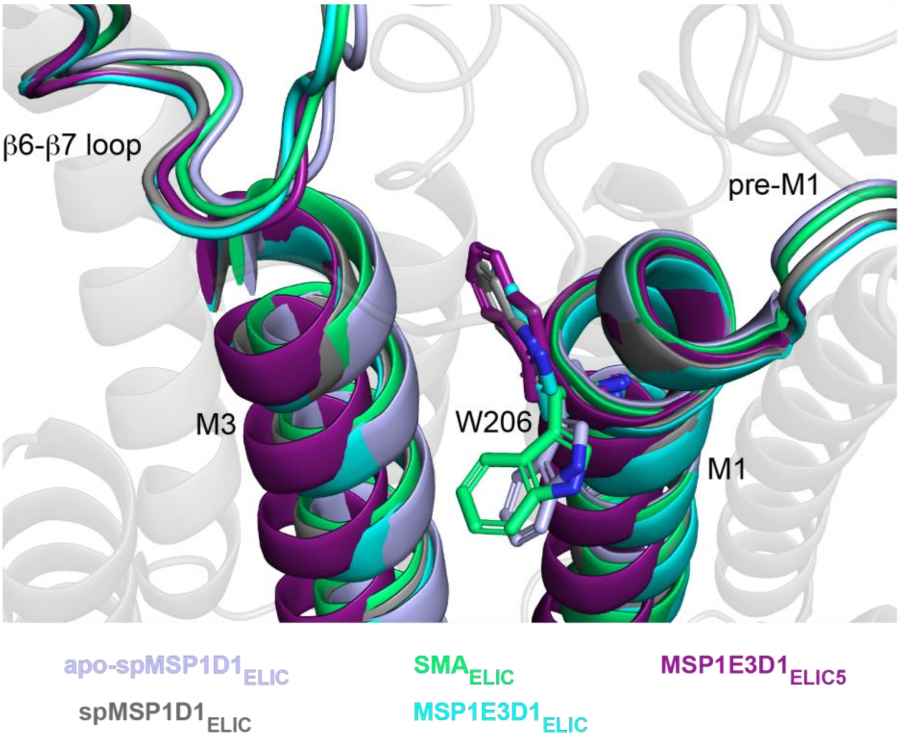

Supplementary Fig. 7: Structural differences in the TMD and ECD-TMD interface of ELIC in different nanodiscs. Image is a global superposition of structures highlighting the β6-β7 loop, pre-M1, and top of M1 and M3. W206 is shown in stick format. W206 faces the membrane when it is pointing down in the image (apo-spMSP1D1$_{ELIC}$ and SMA$_{ELIC}$), and faces an intersubunit groove between M1 and M3 when it is pointing up in the image (MSP1E3D1$_{ELIC}$, spMSP1D1$_{ELIC}$ and MSP1E3D1$_{ELIC5}$). The structure of SMA$_{ELIC}$ more closely resembles apo-spMSP1D1$_{ELIC}$ compared to the other agonist-bound structures.

<u>Supplementary Figure 8</u>

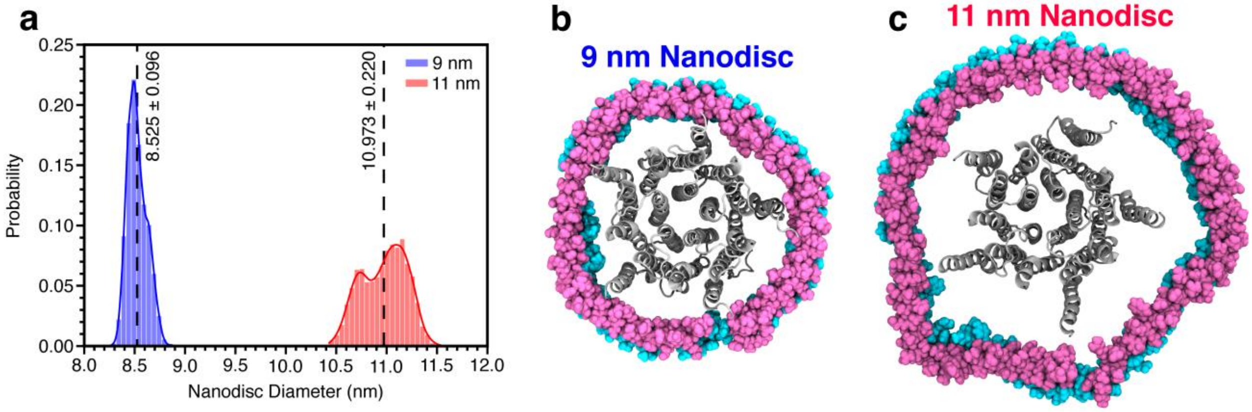

<u>Supplemental Fig. 8:</u> Nanodisc diameters from the MD simulations. (a) The nanodisc diameter for each simulation condition is shown as a histogram with diameters from the 9 nm nanodisc simulations shown in blue and diameters from the 11 nm nanodisc simulations shown in red. The data represents an aggregate from three independent simulations over the last 250 ns of each simulation. The average across all three independent simulations is shown as a vertical dashed line (black) for each condition. The equilibrated nanodiscs containing ELIC are shown for both the 9 nm **(b)** and 11 nm **(c)** simulation conditions. The MSP are shown as cyan and pink van der Waals surface and the ELIC TMD is shown in gray (with the ECD removed for clarity). Lipids, water, and ions have been removed to demonstrate the proximity of ELIC to the MSP.

Supplementary Figure 9

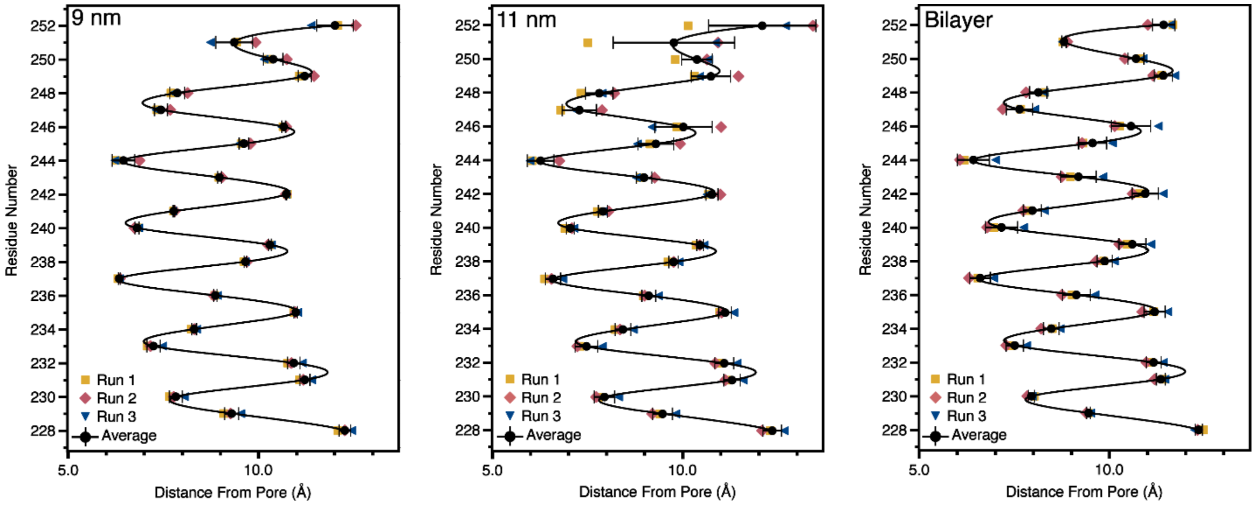

Supplemental Fig. 9: M2-pore axis distance from the MD simulations. Plots of the distance from the α-carbon to the pore axis are shown for each residue of M2 across all simulation conditions (left, 9 nm nanodisc; middle, 11 nm nanodisc; right, planar bilayer). The average from each independent run and the average across all three simulations are shown as separate data points. The error bar represents the standard deviation across the three independent replicates.

Supplementary Figure 10

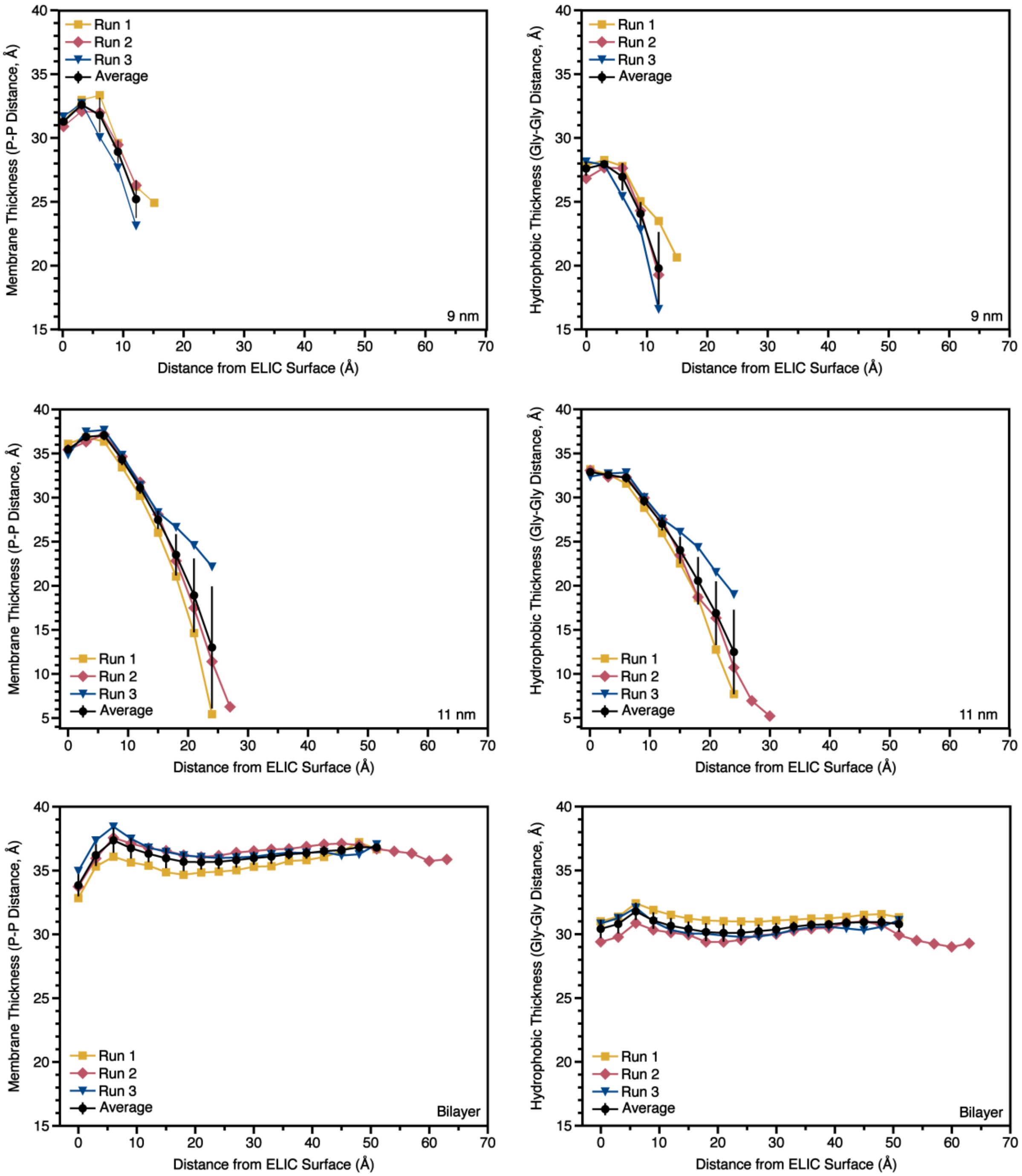

Supplemental Fig. 10: Membrane thickness from the MD simulations. Plots for membrane thickness (left) and hydrophobic thickness (right) are shown for each simulation condition (top, 9 nm nanodisc; middle, 11 nm nanodisc; bottom, planar bilayer). The average thickness across the last 250 ns is shown for each independent replicate as well as the average across the three replicates. The error bars represent the standard deviation across the three independent replicates.
