# Supplementary material for: Lipid nanodisc scaffold and size alter the structure of a pentameric ligand-gated ion channel
Source: Nat Commun. 2024 Jan 2;15:25. doi: 10.1038/s41467-023-44366-w (PMC10762164; doi:10.1038/s41467-023-44366-w)
Supplement: Supplementary file 3 — Reporting Summary [file 41467_2023_44366_MOESM3_ESM.pdf]

nature portfolio

Corresponding author(s): Wayland W. L. Cheng

Last updated by author(s): Nov 27, 2023

Reporting Summary

Nature Portfolio wishes to improve the reproducibility of the work that we publish. This form provides structure for consistency and transparency in reporting. For further information on Nature Portfolio policies, see our Editorial Policies and the Editorial Policy Checklist.

Statistics

For all statistical analyses, confirm that the following items are present in the figure legend, table legend, main text, or Methods section.

n/a	Confirmed	
☐	☒	The exact sample size (*n*) for each experimental group/condition, given as a discrete number and unit of measurement
☐	☒	A statement on whether measurements were taken from distinct samples or whether the same sample was measured repeatedly
☒	☐	The statistical test(s) used AND whether they are one- or two-sided<br>*Only common tests should be described solely by name; describe more complex techniques in the Methods section.*
☒	☐	A description of all covariates tested
☒	☐	A description of any assumptions or corrections, such as tests of normality and adjustment for multiple comparisons
☐	☒	A full description of the statistical parameters including central tendency (e.g. means) or other basic estimates (e.g. regression coefficient) AND variation (e.g. standard deviation) or associated estimates of uncertainty (e.g. confidence intervals)
☒	☐	For null hypothesis testing, the test statistic (e.g. *F*, *t*, *r*) with confidence intervals, effect sizes, degrees of freedom and *P* value noted<br>*Give P values as exact values whenever suitable.*
☒	☐	For Bayesian analysis, information on the choice of priors and Markov chain Monte Carlo settings
☒	☐	For hierarchical and complex designs, identification of the appropriate level for tests and full reporting of outcomes
☒	☐	Estimates of effect sizes (e.g. Cohen's *d*, Pearson's *r*), indicating how they were calculated

Our web collection on statistics for biologists contains articles on many of the points above.

Software and code

Policy information about availability of computer code

Data collection	1. Cryo-EM image acquisition: EPU 2.12.1.2782 and 3.1.0.4506REL<br>2. MD simulations: NAMD2.14
Data analysis	1. Single particle analysis: Relion 3.1 and CryoSPARC 3.3.2<br>2. Motion correction: MotionCor2<br>3. CTF estimation: GCTF v1.06<br>4. Model building: COOT 0.9.6<br>5. Structure refinement: Phenix 1.19.2<br>6. 3D volume visualization and image preparation: PyMOL 2.5.2 and ChimeraX 1.6.1<br>7. Analysis scripts for MD simulations: VMD 1.9.4a55 (running Tcl version 8.5.6), Python 3.6.8, In-house code available at GitHub (https://github.com/mjarcario/NanodiscAnalysis), Zenodo (10.5281/zenodo.10214906) and Dryad (10.5061/dryad.z8w9ghxk5).

For manuscripts utilizing custom algorithms or software that are central to the research but not yet described in published literature, software must be made available to editors and reviewers. We strongly encourage code deposition in a community repository (e.g. GitHub). See the Nature Portfolio guidelines for submitting code & software for further information.

Data

Policy information about availability of data

All manuscripts must include a data availability statement. This statement should provide the following information, where applicable:

- Accession codes, unique identifiers, or web links for publicly available datasets
- A description of any restrictions on data availability
- For clinical datasets or third party data, please ensure that the statement adheres to our policy

The cryo-EM maps have been deposited in the Electron Microscopy Data Bank (EMDB) under accession codes EMD-28829 (SMAELIC), EMD-28830 (saposinELIC), EMD-28831 (spMSP1D1ELIC), EMD-28832 (apo-spMSP1D1ELIC), EMD-41673 (spNW15ELIC), and EMD-41672 (spNW15ELIC5). The structural coordinates have been deposited in the RCSB Protein Data Bank (PDB) under the accession codes 8F32 (SMAELIC), 8F33 (saposinELIC), 8F34 (spMSP1D1ELIC), 8F35 (apo-spMSP1D1ELIC), 8TWZ (spNW15ELIC), and 8TWV (spNW15ELIC5). The MD simulation data from reduced trajectories are deposited in Dryad (10.5061/dryad.z8w9ghxk5).

Research involving human participants, their data, or biological material

Policy information about studies with human participants or human data. See also policy information about sex, gender (identity/presentation), and sexual orientation and race, ethnicity and racism.

Reporting on sex and gender	N/A
Reporting on race, ethnicity, or other socially relevant groupings	N/A
Population characteristics	N/A
Recruitment	N/A
Ethics oversight	N/A

Note that full information on the approval of the study protocol must also be provided in the manuscript.

Field-specific reporting

Please select the one below that is the best fit for your research. If you are not sure, read the appropriate sections before making your selection.

☒ Life sciences ☐ Behavioural & social sciences ☐ Ecological, evolutionary & environmental sciences

For a reference copy of the document with all sections, see nature.com/documents/nr-reporting-summary-flat.pdf

Life sciences study design

All studies must disclose on these points even when the disclosure is negative.

Sample size	For each cryo-EM structure, data were collected to yield final particle counts ranging from 27,201 to 128,454 and such sample sizes were sufficient to produce maps with global resolution ranging from 3.1 to 3.7 angstroms. For the MD simulations, three independent 500 ns simulations were performed. This was deemed sufficient because the properties being measured showed a normal distribution in each replicate suggesting equilibrated behavior, and the results were reproducible across replicates.
Data exclusions	No data were excluded.
Replication	All three replicates for the MD simulations in the three tested conditions were constructed independently, and all attempts at replication were successful. A single cryo-EM structure was obtained for each condition.
Randomization	For the cryo-EM studies, each structure was derived from single biological samples. For the MD simulations, the starting model for each replicate was constructed using the same ELIC structure and different nanodisc or lipid bilayer conditions. Therefore, random allocation of samples or controlling of covariates are not relevant to this study.
Blinding	The experimenter was not blinded to which sample belonged to which condition for the cryo-EM or MD simulation data because this would not impact the analysis of the results.

Reporting for specific materials, systems and methods

We require information from authors about some types of materials, experimental systems and methods used in many studies. Here, indicate whether each material, system or method listed is relevant to your study. If you are not sure if a list item applies to your research, read the appropriate section before selecting a response.

Materials & experimental systems

n/a	Involved in the study
x	Antibodies
x	Eukaryotic cell lines
x	Palaeontology and archaeology
x	Animals and other organisms
x	Clinical data
x	Dual use research of concern
x	Plants

Methods

n/a	Involved in the study
x	ChIP-seq
x	Flow cytometry
x	MRI-based neuroimaging
